# Alterations in circulating markers in HIV/AIDS patients with poor immune reconstitution: Novel insights from microbial translocation and innate immunity

**DOI:** 10.3389/fimmu.2022.1026070

**Published:** 2022-10-17

**Authors:** Qing Xiao, Fengting Yu, Liting Yan, Hongxin Zhao, Fujie Zhang

**Affiliations:** ^1^ Beijing Ditan Hospital, Capital Medical University, Beijing, China; ^2^ Clinical Center for HIV/AIDS, Capital Medical University, Beijing, China; ^3^ Infectious Disease Department, Sichuan Provincial People’s Hospital, University of Electronic Science and Technology of China, Chengdu, China

**Keywords:** human immunodeficiency virus (HIV), acquired immure deficiency syndrome (AIDS), poor immune reconstitution (PIR), plasma markers, fecal markers, innate immunity, microbial translocation

## Abstract

After long-term anti-retroviral therapy (ART) treatment, most human immunodeficiency virus (HIV)/Acquired Immure Deficiency Syndrome (AIDS) patients can achieve virological suppression and gradual recovery of CD4^+^ T-lymphocyte (CD4^+^ T cell) counts. However, some patients still fail to attain normal CD4^+^ T cell counts; this group of patients are called immune non-responders (INRs), and these patients show severe immune dysfunction. The potential mechanism of poor immune reconstitution (PIR) remains unclear and the identification of uniform biomarkers to predict the occurrence of PIR is particularly vital. But limited information is available on the relationship between circulating markers of INRs and immune recovery. Hence, this review summarises alterations in the intestine microbiota and associated markers in the setting of PIR to better understand host-microbiota-metabolite interactions in HIV immune reconstitution and to identify biomarkers that can predict recovery of CD4^+^ T cell counts in INRs.

## 1 Introduction

**Figure 1:**
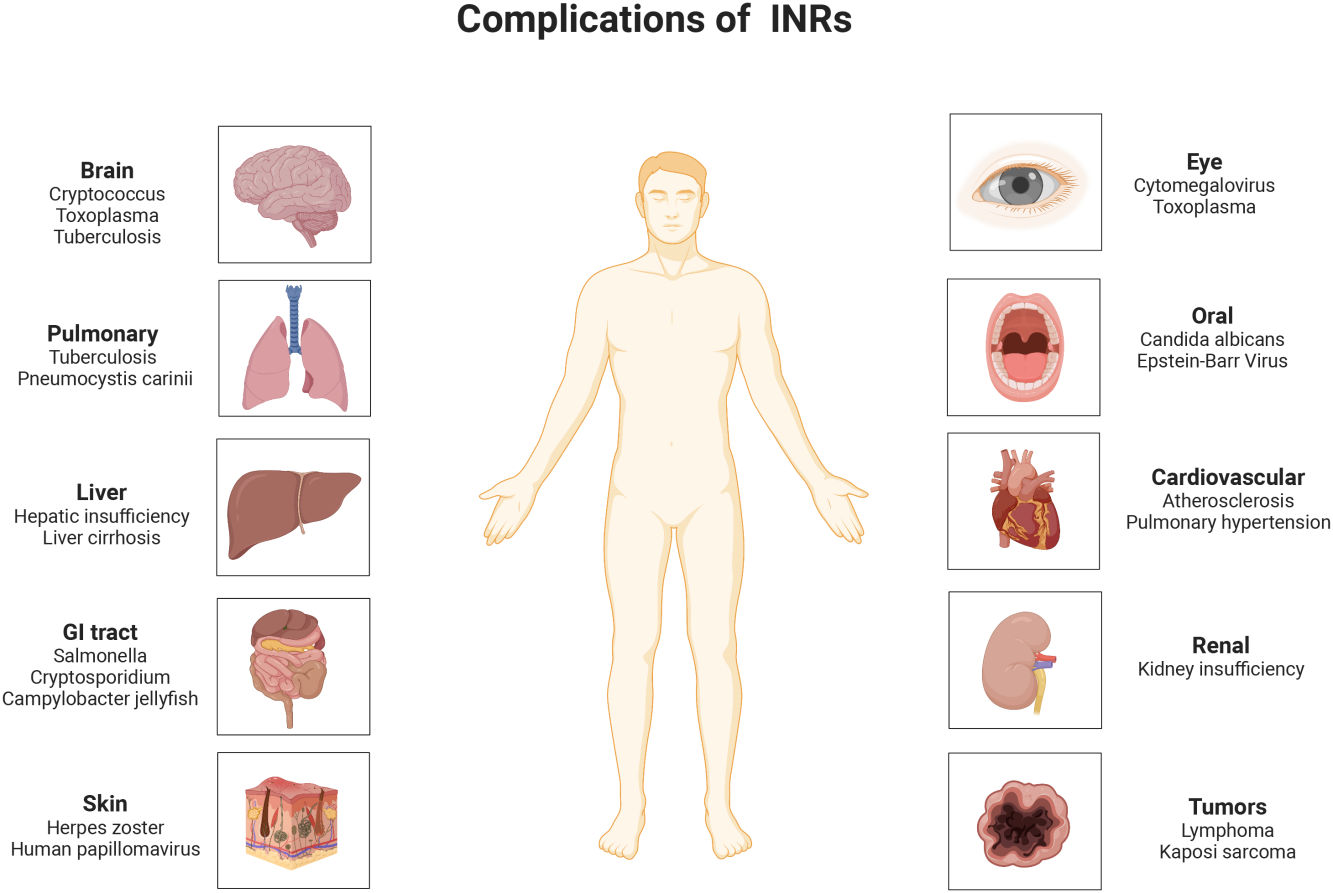
Opportunistic infections and diseases that are likely to complicate in INRs. INRs, immune non-responders.

With the widespread use of anti-retroviral therapy (ART), the viral load (VL) of most human immunodeficiency virus (HIV)/Acquired Immure Deficiency Syndrome (AIDS) patients has been controlled, and peripheral blood CD4^+^ T-lymphocyte (CD4^+^ T cell) counts have returned to relatively normal levels, but approximately 15-30% of ART-treated people living with HIV (PLWH) still have low CD4^+^ T cell counts despite adequate control of viral replication (1, 2). People who suffer from this poor immune reconstitution (PIR) are called immune non-responders (INRs) (3). Persistently low CD4^+^ T cell counts can not only accelerate the progression of Acquired Immune Deficiency Syndrome (AIDS), but also accompany a high mortality rate from AIDS and non-AIDS-related illnesses, causing substantial difficulties in the management of infected individuals (4) (**Figure 1**). There are no universal criteria for PIR. The Department of Health and Human Services (DHHS) defined that HIV/AIDS patients with CD4^+^ T cell count still below 350 or 500 cells/µl after 4-7 years of ART are considered to be INRs (5).

Previous studies examining respiratory fungal communities in HIV/AIDS patients with lung disease have found a strong relationship between lung microbiome and immune status. As CD4 counts decreased, the species diversity of lung bacteria increased but the number declined. At the same time, the quantity and variety of fungus grew as one’s immunological condition deteriorated (6). The primary hallmark of HIV infection is the loss of CD4^+^ T cells. This is most evident in the gut-associated lymphoid tissue (GALT), which houses the majority of lymphocytes in the body (7). Even in the presence of ART, this failed recovery of immune ecological function is associated with an increased risk of microbial translocation, triggers of immune activation, and low-grade chronic inflammation (8, 9). In addition to the intestinal flora, its metabolites and other relevant markers regulate important host activities, such as energy metabolism, intercellular communication, and host immunity (10). Although the pathogenesis of PIR is unclear, it may involve peripheral inflammation, intestinal epithelial impairment, immune damage, and intestinal homing of aberrant immune cells (11). Host metabolic factors are associated with poor CD4^+^ T cell recovery in HIV. It has been found that adipose tissue may affect peripheral CD4^+^ T cell recovery and that excessive activation of CD4^+^ T cell glycolysis may lead to CD4^+^ T cell depletion in HIV infection, and these changes may be reflected in circulating metabolite profiles (12, 13).

Intestinal “translocation” of bacteria or other microorganisms refers to the non-physiological passage of gastrointestinal flora through the intestinal epithelial barrier and lamina propria, eventually reaching local mesenteric lymph nodes, and from there to the body circulation (14). Under normal conditions, translocated microorganisms and microbial products are phagocytosed in the lamina propria and mesenteric lymph nodes (15). However, if the host immune system is compromised, these defense mechanisms may fail, allowing bacteria to escape and survive in distant extra-intestinal sites (16). The intestinal flora and the immune system interact and influence each other. The intestinal flora is closely related to the development of T helper 17 (Th17) cells and plays a crucial part in maintaining the integrity of the intestinal mucosa and the function of the intestinal mucosal barrier (17). The intestinal flora promotes the development of intestinal B cells and protects the intestinal mucosa from exogenous pathogens (18). At the same time, intestinal flora can induce the differentiation of immunosuppressive Treg cells and down-regulate the immune response of the body (19), and can also regulate the immune function of NK cells. Most importantly, intestinal flora can promote tolerogenic differentiation of T cells and enhance the immune tolerance of the intestinal mucosa.

Due to the harmful impact of PIR and the complexity of the underlying mechanisms, it has become essential and urgent to shed light on some biomarkers that reflect immune activation and persistent low-grade inflammation in PLWH on ART (20). However, limited information is available on the relationship between circulating markers and immune recovery in ART-treated PLWH. Meanwhile, with the progression of science and technology, the utilization of body fluids as a sample source to obtain relevant disease information is a breakthrough after high-throughput sequencing technology was applied to molecular diagnosis, which has the advantages of low cost, low adverse effects, and repeatable sampling (21). Therefore, we conducted this review to outline the variations in circulating biomarkers and the link between these markers and immune recovery in INRs, which may help to understand and monitor immune recovery after ART initiation in PLWH.

## 2 Intestinal flora disorders in INRs

**Figure 2:**
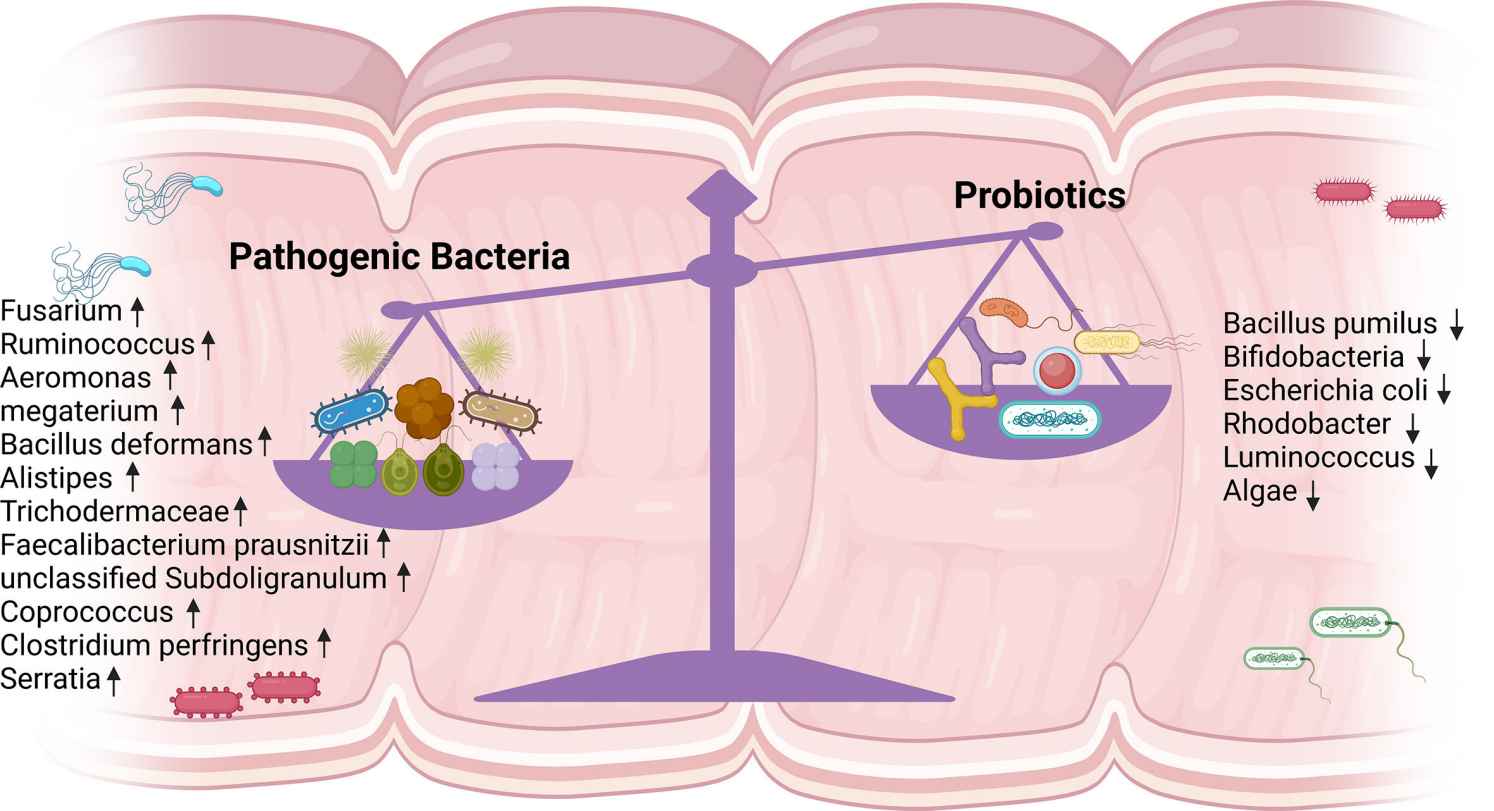
Altered intestinal flora in INRs. A noticeable increase in pathogenic bacteria and a significant decrease in probiotics were detected. INRs, immune non-responders.

HIV infection alters the composition of the intestinal flora non-specifically and diminishes its abundance, characterized by a rise in pathogenic bacteria and a fall in beneficial bacteria, dominated by the absence of bifidobacteria, anaerobic vibrios, clostridia, and Akkermansia (7). One study found that fecal flora alpha diversity declined in PLWH and was independently associated with the patients’ immune status, and that patients with lower CD4^+^ T cell counts had decreased alpha diversity, suggesting that remodeling the intestinal flora may restore immune function in PLWH (22). Microbial diversity plays a crucial role in host immune homeostasis, and the introduction of ART could not fully restore the diversity of the gut microbiota (23). The metagenomic sequencing results of Yirui Xie’s team showed that the INRs and IRs could not be fully restored from the dysregulated gut microbiota in HIV infection. This was demonstrated by the relative greater abundance of Fusarium, Ruminococcus, and Aeromonas megaterium; while Escherichia coli, Algae, Bifidobacterium, Rectobacterium, and Rhodobacter were less abundant in IRs and INRs than in uninfected controls (UCs) (24). Esther Merlini et al. observed that INRs were deficient in probiotic-like lactic acid bacteria both before and after treatment, which was associated with the failure of ART to control microbial translocation of polymicrobial flora in the peripheral blood circulation, and the polymicrobial flora did not change substantially in any way as a result of treatment (25). Edda Russo et al. also detected a rise in algae and a reduction in bacteria in the feces of INRs (26). Danfeng Lu et al. reported lower levels of luminococci in INRs than in IRs and observed differences in alpha diversity between the two groups (27). Judit Villar-García et al. revealed significantly higher inflammatory markers in INRs and more Trichodermaceae and Bacillariidae families in the intestinal flora than in IRs (28). And Gabriella d’Ettorre et al. conducted 48 weeks of probiotic supplementation in INRs and noticed a decrease in both inflammatory markers and T cell activation levels. Even if viral replication is effectively suppressed, further research is needed to restore the diversity of the intestinal flora in PLWH (29). Wei Lu et al. found that Faecalibacterium prausnitzii, unclassified Subdoligranulum sp. and Coprococcus were enriched in INRs. These species were butyric acid-producing and strongly correlated with CD4^+^ T cell counts and T cell immune activation (30). Soo Ching Lee et al. found a significant enrichment of Clostridium perfringens in INRs and a negative correlation between Clostridium perfringens abundance and CD4^+^ T cell count but a positive correlation with CD4^+^ T cell activation and CD4 Treg cell count (31). Krystelle Nganou-Makamdop et al. found that the ratio of Serratia to other bacterial genera changed rapidly following ART. The high ratio in the first year led to inflammation and the first wave of immune reconstitution. In the second year, systemic T cell homeostasis was restored as a result of innate cytokine downregulation and a decrease in Serratia abundance (32). In summary, gut flora may be a breakthrough for HIV immune reconstitution, and detecting circulating flora metabolites and other related markers is probably the most direct way to observe it (**Figure 2**).

## 3 Fecal markers

The intestinal microecology is a critical part of the body’s immune system, with intestinal epithelial cells, intestinal flora, and immune cells interacting with each other to maintain the balance of the intestinal microecology (33). HIV infection causes CD4^+^ T cell depletion, which primarily affects the intestine, severely damages the intestinal epithelium, and enables microbial translocation. Additionally, the modification can activate innate and adaptive immune responses in the blood and gut. Thus, long-term immune activation induces low-grade inflammation in the gut and systemically, resulting in enteropathy (34, 35). Immune activation, pro-inflammatory cytokines, biomarkers of gut damage, microbial translocation, and systemic inflammation are reduced after ART initiation, but remain higher than in uninfected controls (36, 37).

### 3.1 Short-chain fatty acids

Short-chain fatty acids (SCFA) are vital bacterial metabolites that regulate the production of immune mediators for the repair and maintenance of epithelial integrity (38). In addition, SCFA regulates T cell activity and reduces the overexpression of histone deacetylases, such as butyric and valeric acid (39). SCFA is an important connection between the immune system and the microbiome, essential for maintaining homeostasis in the gut, and ultimately plays a role in HIV infection (40). Edda Russo et al. observed variations in the composition of fecal microbiota between IRs and INRs. Fecal isobutyric acid, isovaleric acid, and 2-methylbutyric acid were more significantly increased in IRs, compared with INRs; and they were highly expressed in both groups than in uninfected controls (26). However, they did not change dramatically in the blood. Changes in the gut flora system of PLWH receiving ART may be a consequence and potential cause of systemic immune recovery.

### 3.2 Cytokines

HIV infection is accompanied by an increase in circulating interleukin-18 (IL-18) and a concomitant decrease of its antagonist interleukin-18 binding protein (IL-18BP). HIV infection is also coupled with intestinal inflammation and a loss of intestinal integrity, contributing to increased intestinal permeability and microbial transport. Ossama Allam et al. discovered increased IL-18, IL-1β, and IL-8 in intestinal biopsies of PLWH, which were not related to the ability to reconstitute immunity (41). Mariana Del Rocio Ruiz-Briseño et al. also observed significantly higher levels of IL-1β, IL-8, and IL-18 in the feces of all PLWH treated with ART, but no significant difference in the levels of pro-inflammatory factors in the feces of INRs and IRs (42).

### 3.3 Intestinal inflammation-associated protein

HIV infection is characterized by the depletion of T helper cell 17 cells (Th17 cells), influencing the development of antimicrobial peptides, mucosal regeneration, and neutrophil recruitment. Two key enzymes secreted by neutrophils are fecal calprotectin and lactoferrin, both of which are non-invasive indicators of intestinal inflammation and associated with a variety of gastrointestinal inflammatory diseases (43–45). Mariana Del Rocio Ruiz-Briseño et al. showed that calprotectin and lactoferrin levels were both increased in PLWH and that 17% of the INRs and 5.6% of the IRs exceeded the threshold for lactoferrin associated with intestinal inflammation (5.6 µ g/g in feces). Moreover, 50% of INRs showed elevated lactoferrin and calcineurin concentrations, and low CD4^+^ T cell counts were associated with fecal calcineurin levels (42). When fecal calprotectin levels in HIV-positive children receiving ART were evaluated in another study from Uganda, these kids had excessive quantities of the protein, which was correlated with the development of the illness (46). Changes in gut homeostasis may manifest as activation of the innate immune system and systemic T cells, both of which may contribute to chronic inflammation. Lack of immune rebuilding may be associated with persistent immune activation and inflammation, particularly in the intestinal environment.

## 4 Plasma markers

The destruction of the intestinal mucosa in PLWH causes translocation of the flora and the continuous entry of inflammatory mediators into circulation, creating a chronic immune activation (47). The persistence of low virus levels in their viral reservoirs, such as plasma and monocytes, despite virological suppression with ART, may have contributed to the ongoing immune activation. Such low levels of viremia are more prevalent in patients with PIR (48). Sustained immune activation may impair the initial T cell pool and lead to CD4^+^ T cell depletion (49). Immune activation is higher in INRs and increased T cell activation is predictive of disease progression in HIV infection (50–53). Biomarkers, such as immune activation, inflammation, and flora displacement in serum, are among the main features of HIV infection and disease progression. Serum samples are easily accessible, easy to store and monitor, and more acceptable to patients (54, 55).

### 4.1 Soluble immune mediators

#### 4.1.1 Soluble CD14

Soluble CD14 (sCD14), a ligand for bacterial Lipopolysaccharide (LPS) (56), is detached with the activation of monocytes and macrophages. sCD14 is considered a soluble marker of microbial translocation, inflammation, and innate immune activation, and is associated with all-cause mortality in HIV infection (57, 58). PLWH have been found to have higher levels of sCD14 than UCs (59, 60). A multinational prospective study detected systemic inflammation in African HIV patients with controlled viral loads, and found that plasma sCD14 and C-reactive protein (CRP) concentrations were negatively correlated with subsequent recovery of CD4^+^ T cell counts during ART (61). Gema Méndez-Lagares et al. also revealed that ART did not normalize sCD14 levels in PLWH. Instead, compared to UCs, PLWH had significantly higher plasma sCD14 levels before ART initiation, one year after ART initiation, and five years after ART initiation. In addition, baseline CD4^+^ T cell counts were negatively correlated with baseline sCD14 levels (62). Also, Richard M Dunham et al. discovered that plasma sCD14 levels were significantly higher in INRs than in UCs, but the difference between INRs and IRs was insignificant. And sCD14 levels were negatively correlated with peripheral CD4^+^ T cell levels, a relationship that appeared to be more robust when considering INRs alone (63). Because monocytes/macrophages of neonatal or cord blood origin are highly replicative and more infectious for HIV, the degree of monocyte/macrophage activation and resulting inflammation in perinatally infected children may differ from that in infected adults (64, 65). In HIV-infected children, LPS and sCD14 levels were also increased and persisted even after viral control, and lymphocyte activation was improved by ART (66).

In summary, systemic inflammation and monocyte/macrophage activation were associated with limited CD4^+^ T cell recovery during ART. Long-term innate immune activation may contribute to an HIV-associated inflammatory state, and reduced inflammation may improve CD4^+^ T cell recovery during ART (**Table 1**).

**Table 1:** Overview of studies on plasma levels of sCD14 in INRs.

Author	Research Centers	INRs vs. UCs	IRs vs. UCs	INRs vs. IRs	Relationship between sCD14 and CD4 counts	Ref.
Richard M. Dunham	SCOPE or OPTIONS queue from UCSF, San Francisco, USA	+	ns	NM	–	(63)
Carey L. Shive	Cleveland University Hospital, USA	+	ns	ns	NM	(67)
Xiao-Peng Dai	The Fifth Medical Centre of the General Hospital of the Chinese People’s Liberation Army, China	+	+	+	NM	(68)
Mariana del Rocio Ruiz-Briseño	University Hospital “Fray Antonio Alcalde”, Guadalajara, Mexico	+	ns	ns	NM	(42)
Esther Merlini	San Paolo Hospital, University of Milan, Italy	NM	NM	+	NM	(25)
Esther Merlini	San Paolo Hospital, University of Milan, Italy	+	+	NM	NM	(69)
Stefanie Kroeze	Eight study sites in Kenya, Nigeria, South Africa, and Uganda	NM	NM	NM	–	(61)

NM, not mentioned; ns, non-significant; +, statistically significant; -, negative correlation; INRs, immune non-responders; IRs, immune responders; UCs, uninfected controls.

#### 4.1.2 Soluble CD163

Soluble CD163 (sCD163), a soluble molecule linked to inflammation and monocyte/macrophage activation, is another hallmark of innate immunity. Following inflammatory stimulation, sCD163 can be detached from macrophages by proteolytic cleavage of sheddase ADAM-17 and is therefore considered a marker of inflammation (70). A multicenter cohort study reported that levels of inflammatory and immune activation biomarkers, including sCD163, declined following ART initiation (71). Some studies have also reported no elevated sCD163 levels in PLWH after ART (72, 73). Stefanie Kroeze et al. have identified in a multinational African cohort of late HIV that monocyte/macrophage activation (sCD163, sCD14), inflammation (C-X-C motif chemokine 10 (CXCL10) and CRP), and microbial translocation (lipopolysaccharide-binding protein (LBP)) were persistently elevated (74), which raised the possibility of inadequate CD4^+^ T cell recovery and virus relapse (61). Rebeccah A McKibben et al. found elevated levels of sCD163, sCD14, and C-C Motif Chemokine Ligand 2 (CCL2) in PLWH receiving treatment and that these metrics were associated with atherosclerosis. This led to the conclusion that monocyte/macrophage activation may increase the risk of cardiovascular disease in PLWH (71).

The studies above have focused on comparing HIV-suppressed patients after ART with UCs, and few studies have focused on plasma sCD163 in INRs. Xiaopeng Dai et al. found that plasma sCD163 levels were shown to be higher in INRs compared with UCs, and that ART boosted sCD163 levels in IRs (68). Additionally, the percentage of platelet-CD4+ T cell aggregates was positively linked with the expression of sCD14 and sCD163, which were connected to an enhanced risk of cardiovascular disease (CVD) and can function as indicators of the evolution of HIV illness (75–77).

### 4.2 Fatty acid metabolites

Shi Qian et al. reported significant differences in levels of more than 30 metabolites among INRs, IRs, and UCs using untargeted metabolomics, with lipids accounting for the majority. Compared with UCs, PLWH had reduced levels of certain sterols, while INRs had higher levels of fatty acids and acyl carotenoids than UCs and IRs. Of these, acylcarnitine was the primary differential metabolite. Myristyl carnitine (MC), palmitoylcarnitine (PC), stearoylcarnitine (SC), and oleoylcarnitine (OC) were significantly elevated in the plasma of INRs, and their expressions were inversely connected to CD4^+^ T cell counts (78).

### 4.3 Amino acid metabolites

#### 4.3.1 Tryptophan

Tryptophan is an essential amino acid metabolized predominantly *via* the kynurenine pathway and indoleamine 2,3-dioxygenase 1 (IDO1) is the rate-limiting enzyme for its catabolism (79). The ratio of plasma concentrations of kynurenine to tryptophan (KTR) was used as an indirect measure of IDO activity (80). Julie C. Gaardbo et al. found that PLWHs retaining high KTR exhibited a lower percentage of naïve T cells and preserved an unfavorable distribution of CD4 and CD8 cells, which was consistent with impaired immune reconstitution. In addition, KTR was positively correlated with immune activation, senescence, and apoptosis (81). An *in vitro* study by Peter Terness et al. also showed that downstream products of tryptophan catabolism have toxic effects on T cell responses, inhibiting T cell proliferation and inducing death (82).

Indole-3-propionic acid (IPA), a tryptophan deamidation product produced by the intestinal microbiota, has anti-inflammatory effects and is down-regulated during HIV infection. IPA is vital for intestinal barrier integrity and immune cell functionality, and reflects impaired homeostasis within the gastrointestinal epithelium (83). Furthermore, reduced plasma IPA levels were associated with reduced gut microbial diversity (84). Therefore, the decrease in circulating IPA in PLWH after ART may be a result of changes in gut microbial activity, dysbiosis, and ongoing low inflammation (85–87). Sofia Nyström et al. found IPA to be one of the most significant metabolites that distinguished PLWH from UCs. Plasma expression of IPA and tryptophan was lower in PLWH than in UCs (88).

#### 4.3.2 Cysteine

Cysteine supplies the redox-active sulfhydryl radicals of glutathione, one of the most significant natural antioxidants (89), and oxidative stress has previously been seen in PLWH (90, 91). Cysteine is necessary for T cell growth, which produces reactive oxygen species (ROS) (92). Thomas R. Ziegler et al. found no differences in amino acid concentrations in plasma of INRs and IRs, but PLWH had significantly lower concentrations of total, essential, branched and sulphur amino acids, and 12 individual amino acids than UCs (93). In contrast, Sofia Nyström et al. found that plasma cysteine levels were higher in HIV patients with rapid immune recovery than those with slow immune recovery. Cysteine levels were also elevated in the HIV rapid immune recovery group, compared to UCs. The rapid immune recovery group also had higher concentrations of several metabolic pathways, including homocysteine degradation, cysteine metabolism, and taurine and hypotaurine etabolism, and there was a significant connection between cysteine levels and CD4^+^ T cell count (88). In short, elevated plasma cysteine levels may contribute to the maintenance of redox homeostasis and rapid recovery of CD4^+^ T cell function, and facilitate effective immune reconstitution after ART.

#### 4.3.3 Glycine and serine

ROS levels impact cellular status, with low levels contributing to cell proliferation and high levels leading to cellular senescence or death (94, 95). When cellular metabolism increases and ROS elevates, serine metabolism can maintain cell survival by synthesizing reductive substances to resist excess ROS (96–98). Glycine and serine metabolism is one of the most prominent changes correlated to HIV, where serine and polysaccharides can affect the proliferation and immune function of CD4^+^ T cells in UCs (99). Serine hydroxymethyltransferase catalyzes the conversion of serine to glycine, which in turn directly regulates methionine and serine to replenish single-carbon metabolism (100). The intricate mechanism of single-carbon metabolism is what indirectly regulates cysteine levels and is crucial for preserving cellular redox equilibrium (101). In PLWH with slow immune recovery, levels of these amino acids were reduced (88). Thus, altered serine and glycine metabolism may aid immune recovery by regulating inflammation-induced oxidative stress in PLWH.

### 4.4 Cytokines

#### 4.4.1 Interleukin-6

Interleukin-6 (IL-6), a multipotent cytokine widely distributed in the human body, is involved in the growth and differentiation of a variety of cells and plays a vital role in the body’s acute phase response and immune response against infection (102). Carey L Shive et al. found that plasma IL-6 and sCD14 levels were higher in INRs than in IRs, and that IL-6 levels were positively correlated with the expressions of T cell exhaustion and senescence markers (67). Richard M Dunham et al. assessed IL-6 mRNA and protein levels in the colon. They found that although IL-6 levels were higher in IRs than in UCs and tended to be higher in INRs than in UCs, there was no statistical difference between IRs and INRs, nor was there a correlation between blood IL-6 levels and colonic IL-6 mRNA levels (63).

#### 4.4.2 Interferon-inducible protein-10

Interferon-inducible protein-10 (IP-10), induced by type I and type II interferons, is involved in transporting immune cells to sites of inflammation and is considered a crucial pro-inflammatory factor in the course of HIV disease. In acute HIV infection, plasma IP-10 levels could predict rapid disease progression and were negatively related to CD4^+^ T cell counts (103). With ART intervention, IP-10 levels were reduced, but not to normal levels. And IP-10 was consistently associated with HIV disease progression (based on CD4^+^ T cell count) during this period, suggesting that IP-10 could be used as an indicator of HIV infection or as a target for HIV therapy (104). Carey L Shive found that IP-10 was elevated in INRs and was accompanied by a higher rate of T cell exhaustion and senescence (67).

#### 4.4.3 Interleukin-7 and interleukin-7 receptor

Interleukin-7 (IL-7) is a cytokine that plays a crucial role in the development, maintenance, and renewal of T lymphocytes (105). Studies have reported elevated plasma circulating IL-7 levels in PLWH and its negative correlation with CD4^+^ T cell counts (106, 107). In addition, the IL-7 level is a reliable indicator of the virological reaction to ART in PLWH (107). Consistent with adults, IL-7 levels were significantly higher in HIV-infected children than in age-matched UCs and were negatively correlated with CD4^+^ T cell percentage, but not absolute CD4^+^ T cell count (108–110). In addition, an elevation in plasma IL-7 has also been reported in INRs, consistent with a decrease in T lymphocytes, which may be associated with temporary support of thymic output or peripheral T cell renewal (106, 111, 112). Rethi and her colleagues demonstrated that T cells isolated from PLWH and cultured in the presence of IL-7 had a survival disadvantage, compared with T cells from UCs, suggesting that reduced responsiveness to IL-7 may play a role in disease progression (113).

Marco Marziali et al. observed increased serum IL-7 and decreased naive and thymic naive CD4^+^ T cells in INRs, which was correlated with reduced IL-7Rα in both cell subsets. In addition, increased immune activation, reduced Treg frequency, and increased amplification of the Vβ family were also detected; suggesting that reduced IL-7Rα expression was linked to sustained immune activation and altered Treg frequency, also partially explaining the low levels of CD4^+^ T cell observed in INRs (114). In conclusion, alterations in the IL7/IL7Rα pathway may influence the elevation of CD4^+^ T cells in PLWH with sustained viral load suppression and thus function in the pathogenesis of PIR.

#### 4.4.4 Interleukin-1α and tumor necrosis factor-α

Interleukin-1α (IL-1α), engaged in the formation of acute and chronic inflammation, can activate T, B, and natural killer (NK) cells, increase the neutrophil number and promote the expression of inflammatory cell adhesion molecules (115). TNF-α binds to two receptors, the type I TNF receptor (TNFRI) and the type II TNF receptor (TNFRII), which initiate apoptosis or necroptosis (116). Danfeng Lu et al. demonstrated that INRs had a poorer ratio of CD4/CD8, a worse nutritional condition, and greater amounts of serum cytokines, including tumor necrosis factor-α (TNF-α), interferon-gamma-inducible protein-10 (IP-10) and interleukin-1α (IL-1α), indicating increased chronic inflammation in INRs. Further, lower CD4^+^ T cell counts in INRs were linked to decreased intestinal flora abundance and higher serum pro-inflammatory cytokines. TNF-α and IL-1α levels were also adversely correlated with CD4^+^ T cell counts (27).

#### 4.4.5 Lipopolysaccharide

Lipopolysaccharide (LPS) can trigger inflammation and immune activation by stimulating toll-like receptor 4 (TLR4) on monocytes and other naturally occurring immune cells and is used as a biomarker for microbial translocation. Soluble CD14 (sCD14) plays an essential role in signaling by transferring LPS to the TLR4 complex. Levels of LPS and sCD14 are commonly correlated with intestinal injury, microbial translocation, and inflammation (34). Mariana Del Rocio Ruiz-Briseño et al. found that LPS levels of UCs, IRs, and INRs all showed high variability, and no statistical significance was observed among the three groups (42). However, Esther Merlini et al. found similarly elevated plasma levels of LPS and its ligand sCD14 in both partial immune responders (PRs) and INRs, and this elevation was not reduced by ART (25). These results suggested that microbial translocation could not be fully controlled by ART and was associated with insufficient CD4^+^ T cell reconstitution.

#### 4.4.6 Hypersensitive C-reactive protein

Hypersensitive C-reactive protein (hsCRP) is an inflammatory biomarker that is widely used in a variety of diseases and this immediate reaction raises the risk of disease progression and cardiovascular disease (95). One study found that INRs displayed significantly higher levels of hsCRP, compared with UCs. Although hsCRP is non-specific, its measurement, together with other immune activation and inflammation-related biomarkers, provides information on the status of systemic inflammation (42). In INRs, increases in hsCRP and sCD14 suggest that low-grade systemic inflammation continues despite virological control, which may affect the body’s ability to restore CD4^+^ T cells. Another study found that 17% of IRs and 33% INRs had hsCRP levels above 3 mg/dL; greater risk of death, cardiovascular disease, and the emergence of opportunistic infections were connected to this threshold (117).

#### 4.4.7 Transforming growth factor-β

Transforming growth factor-β (TGF-β) is an essential anti-inflammatory cytokine that promotes the development of regulatory T cells (118). Carey L. Shive et al. found that plasma TGF-β levels in INRs were low and the proportion of circulating CD4^+^ regulatory T cells was reduced, which may make it challenging to control inflammation. In addition, T cell exhaustion and senescence phenotypes were negatively correlated with TGF-β expression and positively correlated with plasma IP-10 and IL-6 levels (67). Thus, there may be a mechanistic link between low TGF-β levels, reduced CD4^+^ T cell recovery, impaired Treg function, and dysregulated T cell phenotype.

#### 4.4.8 Vascular-cell adhesion molecule

Vascular-Cell Adhesion Molecule (VCAM) is not expressed in the resting state, but mainly on the surface of cytokine-activated vascular endothelial cells, mediating the adhesion of lymphocytes and leukocytes to vascular endothelial cells. Under pathological conditions like inflammation, the number and function of VCAM can be significantly upregulated and involved in tissue damage (119). Rodney K Rousseau et al. discovered that soluble VCAM was the only plasma marker elevated in INRs, in comparison with complete responders (CRs). All other plasma biomarkers, including TNF, sCD14, CRP, intercellular cell adhesion molecule (ICAM), monocyte chemotactic protein-1 (MCP-1), gamma-interferon (IFNγ), I-FABP, D-dimer, IL-8, IL-6, and Kyn/Trp, were not significantly different between INRs and CRs (120).

#### 4.4.9 Others

Regarding intestinal permeability, Mariana Del Rocio Ruiz-Briseño et al. measured serum intestinal fatty acid binding protein (I-FABP) and soluble growth stimulated expression gene 2 protein (sST2) levels. Although no significant differences were detected, both I-FABP and sST2 were elevated in IRs and INRs, which may reflect intestinal injury that exacerbated the inflammatory phenotype in PLWH (42). Soluble TNF receptors (sTNF-RI and sTNF-RII, detached upon TNF binding) were measured in plasma as biomarkers of TNF activity. sTNF-RI and sTNF-RII levels did not differ between IRs and INRs. Levels of sTNF-RII were higher in INRs than in UCs, and were more comparable to those observed in viremic subjects (121).

### 4.5 Fibrosis markers

In HIV early infection, lymphoid tissue fibrosis can cause CD4^+^ T cell depletion and immune system dysfunction, and this process may not fully reverse even if ART is started (122, 123). Hyaluronic acid (HA), a component of the extracellular matrix generated during wound healing, is present in elevated concentrations in fibrotic diseases. C-X-C Motif Chemokine Ligand 4 (CXCL4) concentrations rise in response to pro-fibrotic stimuli, and decreased CXCL4 levels in PLWH may imply that HIV effectively evades immunity (124–127). Pre-ART HA and CXCL4 concentrations were found not to vary according to final immune reconstitution status. In INRs, HA concentrations were higher and CXCL4 concentrations were lower after treatment. In paired pre/post-treatment samples, there was a tremendous increase in HA and a more significant decrease in CXCL4 in INRs, compared with UCs. It is hypothesized that increased circulating HA and lower circulating CXCL4 concentrations due to lymphoid tissue fibrosis were associated with PIR (128).

### 4.6 MicroRNAs

Small endogenous RNAs called microRNAs (miRNAs) control post-transcriptional gene expression. Studies have revealed that due to their chemical stability and anti-RNase action, endogenous circulating miRNAs are reliable blood biomarkers (129–131). Circulating miRNAs in serum may be used as indicators of early HIV infection (132), HIV disease progression (133–135), HIV-associated neurological disorders (136–138), and HIV-related liver injury (139, 140). Yuping Fu et al. recognized that numerous miRNAs were found to be downregulated in INRs, compared with IRs; and the miRNA let-7d-5p was identified as a possible biomarker for INRs (141). In contrast, Junnan Lv et al. found that the expressions of five miRNAs (miR-580, miR-627, miR-138-5p, miR-16-5p, and miR-323-3p) were upregulated in the plasma of INRs, compared with IRs. One year after ART, the expressions of these miRNAs were negatively correlated with the increase in CD4^+^ T cell count and percentage. These five miRNAs were utilized to create a predictive algorithm that could successfully and precisely distinguish INRs from IRs to predict PIR following ART. Furthermore, miR-16-5p might prevent the growth of CD4^+^ T cells by regulating calcium flow (142). These prognostic miRNA biomarkers may enhance the early detection of PIR.

### 4.7 Others

Gut bacterial-derived solutes (GBDSs), such as *p*-cresol sulfate (PCS) and indoxyl sulfate (IS), could impair mitochondrial fitness and thus result in an INR-specific reduction in CD4^+^ T cells; and fecal samples of INRs were enriched with bacterial genera capable of producing PCS (23). Ghneim K et al. found that senescent INRs were associated with higher HIV persistence and were driven by the plasma metabolome and microbes with a rich butyrate/bile acid (BA) (143). BA tolerance is an essential property of intestinal colonizing bacteria (144), and the interactions between BA metabolism and the intestinal microbiota are complex. Accumulating toxic bile acids may lead to inflammation (145), and targeting the BA-microbiota axis may be a potential way to improve HIV immune function (146).

## 5 Limitations of this review

This review also has some limitations. First, there are few studies related to circulating markers in INRs, especially in feces. In addition, the accuracy and potential value of these biomarkers in evaluating CD4^+^ T cell recovery in INRs need to be validated in larger sample cohorts. Finally, due to the difference in sample size, the disparity of diagnostic criteria, and the methodological heterogeneity of the included studies, we did not assess the quality and risk of bias of the included studies as a whole, nor did we perform a meta-analysis of individual biomarkers in this review.

## 6 Conclusions and perspectives

**Figure 3:**
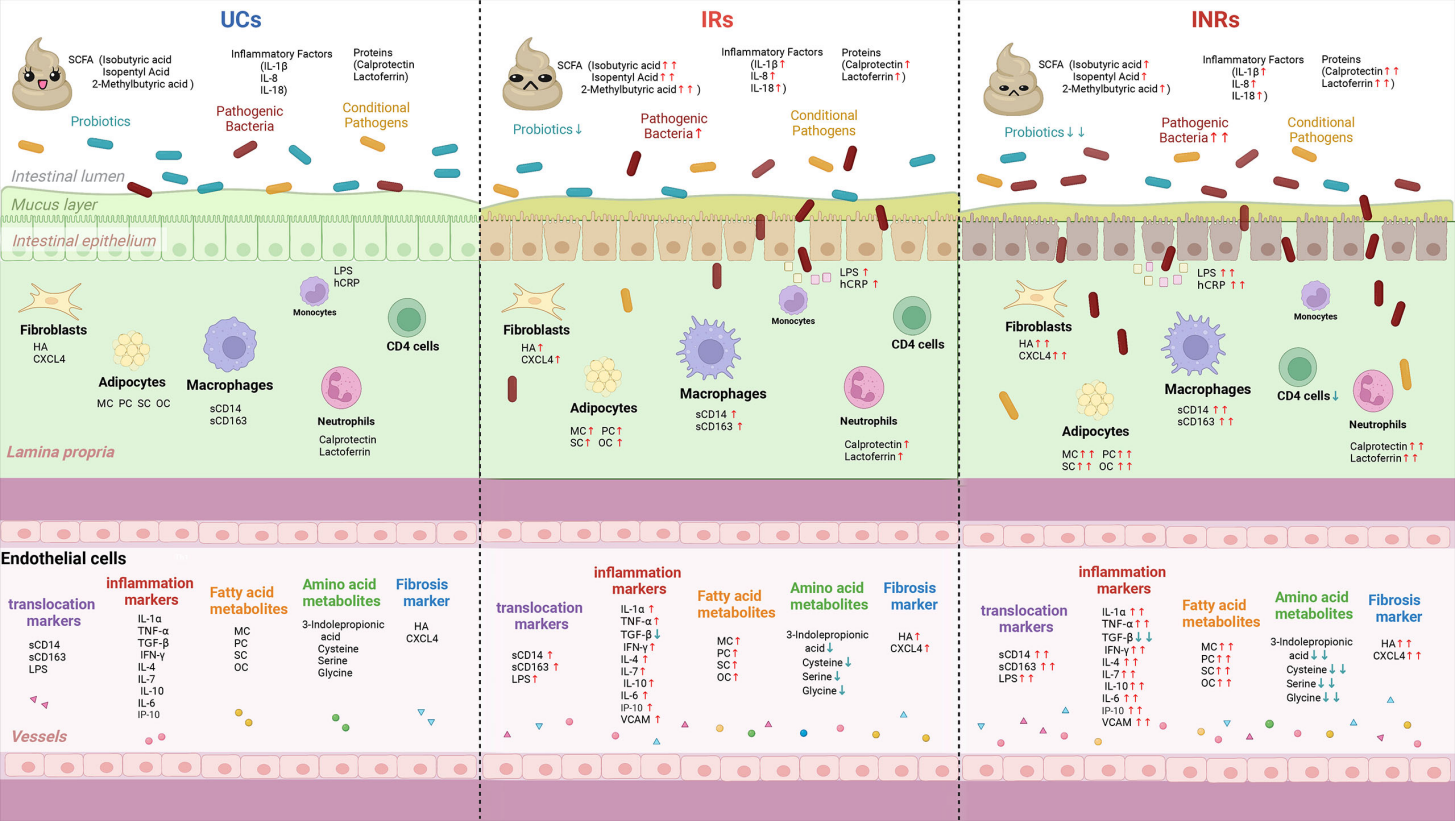
Schematic summary of microbial translocation, immune cells involved, and altered circulating markers in UCs, IRs, and INRs. UCs, uninfected controls; IRs, immune responders; INRs, immune non-responders; SCFA, short chain fatty acids; sCD14, soluble CD14; sCD163, soluble CD163; IL-6, interleukin-6; IL-7, interleukin-7; IL-7R, interleukin-7 receptor; IL-1α, interleukin-1α; IL-1β, interleukin-1β; IL-8, interleukin-8; IL-18, interleukin-18; IP-10, interferon-inducible protein 10; LPS, lipopolysaccharide; hsCRP, hypersensitive C-reactive protein; TGF-β, transforming growth factor-β; VCAM, vascular adhesion molecules; HA, hyaluronic acid; CXCL4, C-X-C motif chemokine ligand 4; MC, myristyl carnitine; PC, palmitoylcarnitine; SC, stearoylcarnitine; OC, oleoylcarnitine; ↑, increase in comparison with uninfected controls; ↓, decrease in comparison with uninfected controls.

ART increases CD4^+^ T cell counts and partially rebuilds the immune system in PLWH, but still fails to resolve microbial translocation or monocyte/macrophage activation, thereby increasing long-term morbidity and mortality in non-AIDS-associated inflammatory conditions (**Figure 3**). Furthermore, we revealed that the systematic study of diverse circulating markers might deepen the understanding of the immunopathogenesis of HIV and provide new tactics for clinical treatment (**Table 2**). Considering the vital role of circulating markers in immune programming and regulation, future studies on changes of gut flora and circulating biomarkers in INRs should be conducted in more detail to elucidate the regulatory mechanisms of the circulating microenvironment on CD4^+^ T cell reconstitution. Overall, this review offers a new perspective on the impact of HIV infection, ART, and the microbiota immune axis on immune recovery at the metabolic level.

**Table 2:** Summary of circulating marker changes in INRs.

Samples	Feces			Plasma						
Classification	SCFA	Cytokines	Intestinal inflammation-associated proteins	Soluble immune mediators	Fatty acid metabolites	Amino acid metabolites	Cytokines	Fibrosis markers	MicroRNA	Others
**Items**	Isobutyric acid ↑	IL-1β↑	Calprotectin↑	sCD14↑	Myristyl Carnitine↑	3-Indolepropionic acid↓	IL-6↑	HA↑	miRNA let-7d-5pi↓	PCS↑
	Isovaleric acid ↑	IL-8↑	Lactoferrin↑	sCD163↑	Palmitoylcarnitine↑	Cysteine↓	IP-10↑	CXCL4↓	miR-580↑	IS↑
	2-Methylbutyric acid ↑	IL-18↑			Stearoylcarnitine↑	Serine↓	IL-7↑		miR-627↑	BA↑
					Oleoylcarnitine↑	Glycine↓	IL-7R↓		miR-138-5p↑	butyrate↑
							IL-1α↑		miR-16-5p↑	
							TNF-α↑		miR-323-3p↑	
							LPS↑			
							hsCRP↑			
							TGF-β↓			
							VCAM↑			

INRs, immune non-responders; SCFA, short chain fatty acids; sCD14, soluble CD14; sCD163, soluble CD163; IL-6, interleukin-6; IL-7, interleukin-7; IL-7R, interleukin-7 receptor; IL-1α, interleukin-1α; IL-1β, interleukin-1β; IL-8, interleukin-8; IL-18, interleukin-18; IP-10, interferon-inducible protein 10; LPS, lipopolysaccharide; hsCRP, hypersensitive C-reactive protein; TGF-β, transforming growth factor-β; VCAM, vascular adhesion molecules; HA, hyaluronic acid; CXCL4, C-X-C motif chemokine ligand 4; PCS, p-cresol sulfate; IS, indoxyl sulfate; BA, bile acid; ↑, increase in comparison with uninfected controls; ↓, decrease in comparison with uninfected controls.

## Author contributions

All authors made intellectual contributions to this work. QX and FZ had the idea for this review, QX wrote the article, and FY, LY, HZ, and FZ supervised the manuscript. All authors contributed to the article and approved the submitted version.

## Funding

This work was supported by the 13th Five-year Plan, Ministry of Science and Technology of China (2018ZX10302-102), Beijing Municipal Administration of Hospitals’ Ascent Plan (DFL20191802), and Beijing Municipal Administration of Hospitals Clinical Medicine Development of Special Funding Support (ZYLX202126). The funders had no role in study design, data collection, analysis, publication decisions, or manuscript preparation.

## Acknowledgments

Thanks to all authors for their contribution to this manuscript.

## Conflict of interest

The authors declare that the research was conducted in the absence of any commercial or financial relationships that could be construed as a potential conflict of interest.

## Publisher’s note

All claims expressed in this article are solely those of the authors and do not necessarily represent those of their affiliated organizations, or those of the publisher, the editors and the reviewers. Any product that may be evaluated in this article, or claim that may be made by its manufacturer, is not guaranteed or endorsed by the publisher.
